# Effects of alcohol hangover on attentional resources during a verbal memory/psychomotor tracking dual attention task

**DOI:** 10.1007/s00213-022-06150-4

**Published:** 2022-05-11

**Authors:** Elizabeth Ayre, Sarah Benson, Harriet Garrisson, Katherine H. M. Cox, Joris C. Verster, Andrew Scholey

**Affiliations:** 1 Centre for Human Psychopharmacology, Swinburne University of Technology, Hawthorn, VIC 3122 Australia; 2 Division of Pharmacology, Utrecht Institute for Pharmaceutical Sciences (UIPS), Utrecht University, Utrecht, 3584 CG The Netherlands; 3 Nutrition Dietetics and Food, Monash University, Clayton, VIC 3800 Australia

**Keywords:** Alcohol hangover, Attention, Memory encoding, Attentional resources, Recognition, Perceived performance

## Abstract

**Background:**

Alcohol hangover (AH) is associated with impaired attention and memory performance. However, whether this effect is related to reduced attentional resources remains unclear.

**Aims:**

A dual-attention paradigm was employed to assess the effects of AH on attentional resources, delayed memory recognition, and the interaction between attentional load and AH. Mental effort and perceived performance during AH and control conditions were also assessed.

**Methods:**

A seminaturalistic, crossover design was used. In total, 25 healthy social drinkers aged 18–35 years, visited the laboratory following a typical night out drinking (Hangover condition) and after alcohol abstinence (control) between 8:30 am and 12:30 pm, with conditions counterbalanced. Attentional load was manipulated via the presence (dual attention) or absence of psychomotor tracking during verbal memory encoding. Perceived mental effort and performance were measured using the NASA-TLX. Participants’ recollected alcohol consumption was used to compute estimated blood alcohol level (eBAC).

**Results:**

Compared with the control visit, AH was associated with reduced recognition accuracy (particularly more false negatives), higher “tracking costs” (poorer accuracy) in the dual attention condition, increased ratings of “mental demand,” “effort,” and “frustration,” and lower ratings of task performance. There was also a significant main effect of attentional load with poorer recognition accuracy and response time in the dual attention condition. There were no significant interaction effects between hangover and attentional load.

**Conclusion:**

These findings suggest that reduced attentional resources contribute to the cognitive deficits associated with AH including impaired memory consolidation. They further suggest that while hungover, participants are aware of these deficits but are unable to compensate.

## Introduction

Alcohol-hangover (AH) is the common after-effect of a single episode of heavy drinking that starts when blood alcohol concentration (BAC) is approaching 0.00% (van Schrojenstein Lantman et al. 2016; Verster et al. 2020b). It involves a combination of adverse mental and physical symptoms that often includes headache, thirst (Penning et al. 2012; Piasecki et al. 2010), fatigue (McKinney and Coyle 2006; Verster et al. 2010), and nausea (Penning et al. 2013; van Schrojenstein Lantman et al. 2016). AH has been associated with impaired cognitive functioning (Gunn et al. 2018; Verster et al. 2014), and was recently included as a separate entity from acute intoxication in the International Classification of Diseases (ICD-11) (World Health Organisation 2021). Impaired cognitive functioning may be a key contributor to annual socioeconomic losses (Prat et al. 2009; Wiese et al. 2000), which have been estimated at 452 million AUD in Australia from absenteeism (Roche et al. 2015), 1.2–1.4 billion GBP in the UK from reduced productivity (Bhattacharya 2019), and 249 billion USD in the USA when factoring total costs (Sacks et al. 2015).

Despite this, research on AH-induced cognitive impairment has gained momentum only in the last decade. This has revealed AH impairments across various domains including information processing (Grange et al. 2016), reward learning (Howse et al. 2018), psychomotor speed (McKinney and Coyle 2004; 2007), and cognitive flexibility (Gunn et al. 2020; Scholey et al. 2019). In relation to attentional processes, several studies have reported impairments in sustained (Howland et al. 2010; McKinney et al. 2012; Rohsenow et al. 2010) and selective attention (Devenney et al. 2019; McKinney et al. 2012), as well as reduced self-reported alertness (Benson et al. 2020; Devenney et al. 2019; McKinney and Coyle 2007; Verster et al. 2003) and concentration (Finnigan et al. 2005; Slutske et al. 2003; Verster et al. 2014). Along with higher ratings of perceived mental effort and demand (Gunn et al. 2020, 2021), this suggests an association with reduced attentional resources during AH.

Not all studies have found impairments in attentional processes during AH, particularly those assessing divided attention. Barring one study that found higher errors during a tracking/reaction-time dual-task (albeit in a small sample (*N* = 5)) (Roehrs et al. 1991), most studies measuring divided attention have reported no AH-related impairments (Chait and Perry 1994; Collins 1980; Collins and Chiles 1980; Devenney et al. 2019; Finnigan et al. 1998, 2005; Lemon et al. 1993; McKinney et al. 2012). The reasons for this are unclear, although they may be somewhat related to several studies being older with relatively low statistical power (Benson et al. 2020; Gunn et al. 2018).

Another possible explanation for the limited findings relates to task selection. Previous studies have commonly used visual divided attention tasks to compare AH and non-AH conditions. These tasks involve participants simultaneously attending to two tasks (a primary task in central vision and secondary task in peripheral vision) on the same computer screen, with central task errors and/or the ability to perceive irregular peripheral stimuli as outcome measures. While these tasks are a widely used psychological tool, they may be problematic for assessing AH effects for two reasons. Firstly, the secondary task has relatively low attentional requirements (Naveh-Benjamin et al. 2005; Scholey et al. 2008). Other studies measuring visual perception have also found limited (Kim et al. 2003) or no related impairments during AH (Stock et al. 2017). In the latter study, the processing of visual information was found to be faster during AH when compared with control conditions (i.e., faster accumulation of visual information was observed through higher drift rates using diffusion modeling). This suggests that the task may not be difficult enough to challenge attentional resource capacity or be sensitive to determining AH-related impairments (Naveh-Benjamin et al. 2005; Scholey et al. 2008).

Secondly, and somewhat related to the first point, in these previous studies, attention was not divided across differing modalities (Stephens et al. 2014). In complex cognitive tasks like driving, both visual and auditory processes need to be active. This increased cognitive load places a higher demand on attentional resources. Although divided attention is not strictly measured per se, AH impairments have been observed in studies measuring complex performance including simulated driving (Alford et al. 2020a; Verster et al. 2014) and flying ability (Yesavage et al. 1994; Yesavage and Leirer 1986). Importantly, these impairments were evident despite a significant increase in self-reported mental effort during AH (Alford et al. 2020a; Verster et al. 2014). We have previously reported AH impairment of multitasking performance using a four-module version of the Purple Multi-Tasking Framework (MTF) (Benson et al. 2020). While overall aggregate performance was impaired, there were no effects on individual modules (mental arithmetic, Stroop, working memory, psychomotor tracking), suggesting that AH impairs performance when attention is divided or where there may be competing cognitive demands.

Other cognitive processes like memory also need available attentional resources to function efficiently. Indeed, a meta-analysis indicated that short and long-term memory processes were impaired during AH, with impairments thought to be due to the effects on memory encoding rather than retrieval (Gunn et al. 2018). As such, Gunn et al. (2018) noted that the subsequent impairments in word recall and/or recognition were observed only when learning and retrieval commonly occurred during AH (McKinney and Coyle 2004; 2007; Verster et al. 2003) and not when learning occurred prior to AH in a non-AH state (Howland et al. 2010).

When considered together, deficits in memory encoding during AH may be exacerbated by reduced attentional resources in conditions where attention is divided. Studies unrelated to AH have adopted dual-attention paradigms to explore this interplay between attentional load and memory finding impairments in both memory recognition and secondary task performance (Anderson et al. 2000; Baddeley et al. 1984; Blanchet et al. 2009; Craik et al. 1996; Iidaka et al. 2000; Naveh-Benjamin et al. 2005; Scholey et al. 2008). In the canonical paradigm, participants are required to track a moving target using the computer mouse while being presented with words to remember via headphones. As words are encoded the ability to track is compromised as attentional resources are diverted to encoding, leading to poorer psychomotor tracking or “tracking cost.” This paradigm is sensitive to both enhanced and impaired tracking and memory. For example, aging is associated with higher tracking costs (Naveh-Benjamin et al. 2005), whereas glucose loading reduced tracking costs (Scholey et al. 2008). These findings have been interpreted as reflecting depleted and higher attentional reserves, respectively.

Reduced multitasking ability in AH may reflect reduced attentional resources. As such, we applied the dual-attention paradigm described above to AH. This was achieved by manipulating attentional load via the presence and absence of psychomotor tracking during the encoding phase of a verbal memory task and comparing (1) tracking costs, (2) delayed memory recognition, and (3) the interaction between attentional load and AH. It was hypothesized that AH would reduce attentional resources resulting in poorer tracking performance, that AH would also reduce memory recognition ability, and that this effect would be significantly worse in the divided attention condition. Secondary aims included comparing mental effort of task performance during AH and control visits, and assessing whether the severity of AH was associated with task performance and mental effort.

## Method

### Design

The current study utilized a seminaturalistic, crossover design with counterbalanced session order. Seminaturalistic designs allow participants to consume alcohol in a familiar environment, on a “typical” night out, and attend testing in the laboratory the following morning when experiencing an AH. Studies using this research design have been found to have high ecological validity, with estimated alcohol consumption being consistent with participants’ normal single-occasion alcohol intake (McKinney et al. 2012; Verster et al. 2014). Seminaturalistic studies have been successful at discerning the effects of AH across various cognitive domains (Devenney et al. 2019; Gunn et al. 2020; Verster et al. 2019). The control session was conducted following 24 h of alcohol abstinence. This study was approved by Swinburne University Human Research Ethics Committee (SUHREC, 2016/061) and conducted in accordance with the declaration of Helsinki.

### Participants

Participants were healthy social drinkers from metropolitan Melbourne, Australia. Participants were recruited via website advertisements (i.e., Facebook, Gumtree Australia), flyers, and the university’s research experience program. Participants were required to meet the following criteria: (1) aged 18–40 years, (2) nonsmoker, (3) experience AH frequently (once a month), (4) no past or current alcohol, (5) drug and (6) psychiatric problems, (7) not taking medications that could interact with alcohol, (8) willing to abstain from illicit drug use for the duration of the study, (9) no liver or renal impairments, and (10) not pregnant or nursing (females). Problematic alcohol use was assessed using the Alcohol Use Disorder Identification Test (AUDIT) (World Health Organisation 2001).

### Cognitive measures

#### Dual-attention task (DATT)

The DATT was created using ePrime 2.0 (Schneider and Zuccoloto 2007), to investigate the associations between AH, attentional resources, and memory recognition. This task was developed from previous studies using the same paradigm (Scholey et al. 2008).

#### Target tracking/memory encoding

For target tracking, participants used a mouse to track a moving target (red dot) around a white computer screen, aiming to keep the cursor as close to the target as possible. The target followed a smooth random path at approximately 6 cm/s, a speed found to be moderately difficult and not produce ceiling effects (Naveh-Benjamin et al. 2005; Scholey et al. 2008). For memory encoding, 20 random and unrelated two- and three-syllable words (e.g., “GARDEN,” “PAPER”) were presented via headphones. The words were drawn from the University of South Florida Word Association, Rhyme and Word Fragment Norms database (Nelson et al. 1998). They were matched on an accessibility index (“a measure of the ease with which a given word comes to mind”) and for written frequency. Each word had a duration of ≤ 1000 ms and was followed by a 2000 ms interstimulus interval. Words were presented at fixed 3000 ms intervals from the start of the previous stimulus. Four groups of word lists were created, with each group consisting of two separate word lists, one presented alone and one in divided attention conditions, for both AH and control conditions. List order was counterbalanced across AH and attention conditions.

To complete the task, participants practiced tracking before listening to one word list in isolation. Participants then completed target tracking while listening to the second list of words simultaneously. The first word was presented 2000 ms after tracking began. Outcomes were the distance between target and cursor in the dual attention condition, which was computed every 100 ms and converted to a “tracking cost” in pixels. Greater distances between the target and cursor indicated higher tracking costs relating to reduced attentional resources.

#### Delayed memory recognition

Word recognition was completed following a fixed delay during which a cognitive multi-tasking battery (MTF; 20-min) and associated questionnaires were completed (results presented in Benson et al. (2020)). Participants were presented with 40 words consisting of 10 each from focused and dual-attention conditions, plus 20 novel distractor words. Participants indicated whether the word was presented in either original list using left and right arrows on the computer keyboard marked “yes” and “no,” respectively. Outcomes were the speed (ms) and accuracy (%) of responses to focused (-tracking), dual-attention (+ tracking) and distractor words. The accuracy of “yes” and “no” responses were also analyzed to determine the proportion of false positives (incorrect “yes” responses) and false negatives (incorrect “no” responses). Lower accuracy percentages indicated a higher proportion of false responding.

#### Pencil and paper scales

*NASA Task Load Index (NASA-TLX; (*Hart and Staveland 1988*)).* The NASA-TLX assessed perceived workload on the DATT. Visual analogue scales flanked by the words “low” and “high” measured six dimensions: mental, physical and temporal demands, performance, effort, and frustration.

*Alcohol-Hangover Severity Scale (AHSS; (*Penning et al. 2013*))*. The 12-item AHSS assesses the severity of commonly reported AH symptoms on an 11-point Likert scale (0 = *absent* to 10 = *extreme*). Items including “thirst”, “fatigue,” and “concentration problems” were added and averaged to indicate an overall AH severity score. The AHSS has been found to have good internal consistency (Cronbach’s *α* = 0.85) and predictive validity (*r* = 0.92) (Penning et al. 2013).

*Single-Item Alcohol-Hangover Severity*. Subjective overall AH severity was also measured using a visual analogue scale. Participants were asked to rate “how severe is your hangover” between 0 (no AH symptoms) and 10 (very severe AH symptoms). Previous research recommends the use of a single-item AH severity question in conjunction with an AH questionnaire that assesses individual AH symptoms (Verster et al. 2020c). A single-item AH severity question reduces the reliance on the AHSS to assess overall AH severity which is prone to bias due to variability in the presence and severity of common versus less common symptoms and the inability to include all possible AH symptoms in the scale (Verster et al. 2020c).

*Alcohol Consumption.* Participants stated the number of standard drinks and hours spent drinking the evening prior to the AH test visit. Australian standard drink sizes (1 unit = 10 g pure alcohol) were provided as a visual reference, with categories including beer, cider, wine, and spirits (Australian Institute of Health and Welfare 2017).

*Estimated BAC (eBAC).* Peak eBAC the evening before the AH visit was calculated using the responses to alcohol consumption questions. As outlined in Benson et al. (2020), eBAC was determined by calculating the average estimated total body water (TBW) of participants using the formulas of Widmark (1981), Watson et al. (1981), Forrest (1986), Seidl et al. (2000), and Ulrich et al. (1987) (males only). The average TBW was submitted to the formula:

$$BAC=\left(G/TBW\right)-\beta \times t$$

where *G* constitutes the amount of alcohol consumed in grams, *β* equals the metabolic rate (g/hr), and *t* is the time of alcohol consumption in hours.

### Procedure

Following an initial telephone screening where eligibility was assessed, participants visited the laboratory at Swinburne University on three occasions where they completed one screening and two test sessions (one AH, one control, counterbalanced). At the screening session, participants provided written informed consent and practiced all cognitive tasks. At the end of the session, participants were randomized, so 50% of participants completed their AH session first. Test visits were arranged around the participants planned drinking activities to minimize disruptions to standard drinking behavior and represent real-life AH experiences. Participants were also advised to consume their usual amount of alcohol and not to consume alcohol for the purpose of the study. Sessions were scheduled 5–14 days apart.

On testing days, participants were asked to refrain from caffeine and to consume the same breakfast. Upon arrival to testing (between 8:30 am and 12:30 pm and held consistent for both visits), compliance to these restrictions was confirmed, and participants were breathalyzed using a regularly calibrated Lion Alcolmeter® SD400PA (Lion Breathalysers Australia Pty Ltd). No participant recorded a BAC reading > 0.00%. Participants then completed tests in the following order: DATT-tracking/memory encoding, cognitive multi-tasking battery (20 min) and associated questionnaires (results presented in Benson et al. (2020)), DATT-word recognition, NASA-TLX, AHSS, questions on the previous evenings sleep (see Benson et al. (2020)), alcohol consumption, and single-item AH severity. Computerized versions of alcohol consumption and AH-related questions were used. At study completion, participants were reimbursed $30 (AUD) to cover out of pocket travel expenses.

### Data treatment and analyses

Analyses were conducted using SPSS version 26 (IBM Corp, Armonk, NY, USA). The initial screening revealed 21 missing values in the DATT tracking/memory encoding task (no other variables were missing data). Out of 1200 recorded distances between the target and cursor (i.e., per 100 ms), six participants were missing two time points, while a further nine participants were missing one. A nonsignificant missing value analysis ensured these values were missing at random (*χ*^*2*^(273) = 266.95, *p* = 0.592) and could be replaced using mean substitution. This was calculated by averaging the distances from time points on either side of each missing value (i.e., 100 ms before and after the missing value) (Tabachnick et al. 2007). Outliers were also detected by scores > 1.5*interquartile range. To retain the sample size, outlying scores were reduced by winsorizing, which did not impact the results presented below (Dixon and Yuen 1974; Tabachnick et al. 2007). This was established by running the analyses with and without outliers and confirming significant results using nonparametric Wilcoxon signed-ranked tests.

For the DATT-tracking/encoding task, the first 2000 ms was removed, as it did not coincide with word presentation or the interstimulus interval. Tracking costs at each 100 ms time point were then averaged over 20 words (1000 ms) and 20 interstimulus intervals (ISI, 2000 ms) to create 30 mean time scores. Averaged tracking costs were analyzed by a two-way (hangover × time) repeated measures ANOVA, with two levels of hangover (AH, control) and 30 levels of time. For delayed recognition accuracy and reaction time, two-way repeated measures ANOVAs with two levels of hangover (AH, control) and two levels of load (-tracking, + tracking) were also adopted. All other variables comparing AH and control conditions were analyzed using paired-sample *t* tests that were two-tailed. To account for dependence in the data, Cohen’s *f* or Cohen’s *d* effect sizes were calculated for all significant tests using Eq. 8 (Morris and DeShon 2002). Where there were violations of normality (i.e., mental and physical demand on the NASA-TLX), effects were assessed using BCa bootstrapped samples with 95% CI. Further measures of association between AH severity, task performance, and mental effort were analyzed by Pearson’s correlation coefficients.

## Results

### Participant characteristics

In total, 36 participants took part in the study. However, eight participants failed to complete testing visits and three participants reported alcohol intake the evening before the control visit. Although one participant scored in the AUDIT range considered to be harmful use of alcohol (i.e. score of 17), an evaluation of the participants drinking history ensured they did not meet our criteria for problematic drinking or potential alcohol use disorder. Demographic characteristics for the final sample of 25 participants (76% female) are presented in Table 1.

**Table 1:** Demographic characteristics for the sample of *n* = 25

	M	SD
Age (years)	25.32	4.32
AUDIT score	7.84	3.54
Drinks consumed	8.15	4.22
Time drinking (hours)	4.23	1.23
eBAC (%)	0.135	0.07

### Alcohol-hangover severity

Overall scores on the AHSS for AH (*M* = 3.86 ± 1.83) compared with control (*M* = 0.70 ± 0.74) were significantly higher (*t*(24) = 8.90, *p* < 0.001, *d* = 2.03, 95% CI [2.43, 3.90]) (for individual item scores, see Benson et al. (2020)). Single-item AH severity for AH (*M* = 4.72 ± 2.36) compared with control (*M* = 0.05 ± 0.16) was also significantly higher (*t*(24) = 10.07, *p* < 0.001 *d* = 3.07, 95% CI [3.71, 5.63]).

### *Tracking accuracy*

**Fig. 1:**
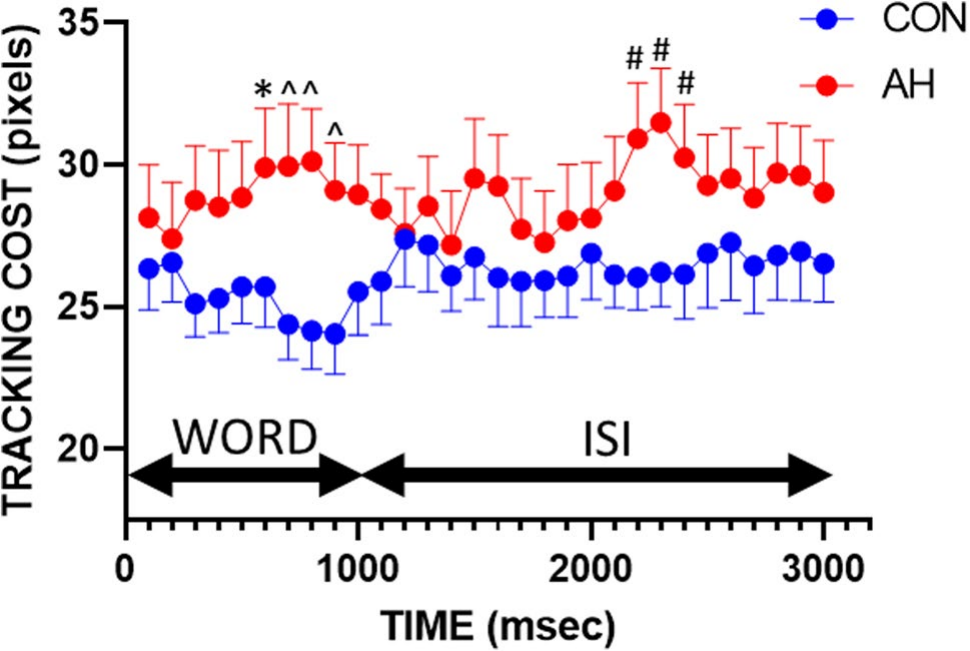
Mean cost to tracking performance in pixels per 100 ms averaged for 20 words and 20 interstimulus intervals. Significant effects of AH are indicated by *p < .05, #*p* < .02, ˄*p* < .01

A significant hangover × time interaction (*F*(29,696) = 1.63, *p* < 0.05, *f* = 0.26) was found on the DATT-tracking/memory encoding task. Pairwise comparisons were used to assess the differences at each 100 ms time point, with significantly further distances between the cursor and target representing a significant “tracking cost.” As shown in Fig. 1, there was a significant cost between control and AH from 700 to 900 ms when a word was present and again from 2200 to 2400 ms during the interstimulus interval. Significant differences were verified using nonparametric paired comparisons (Wilcoxon signed-rank tests), which also resulted in significant effects (*ps* < 0.05).

### Memory recognition

**Fig. 2:**
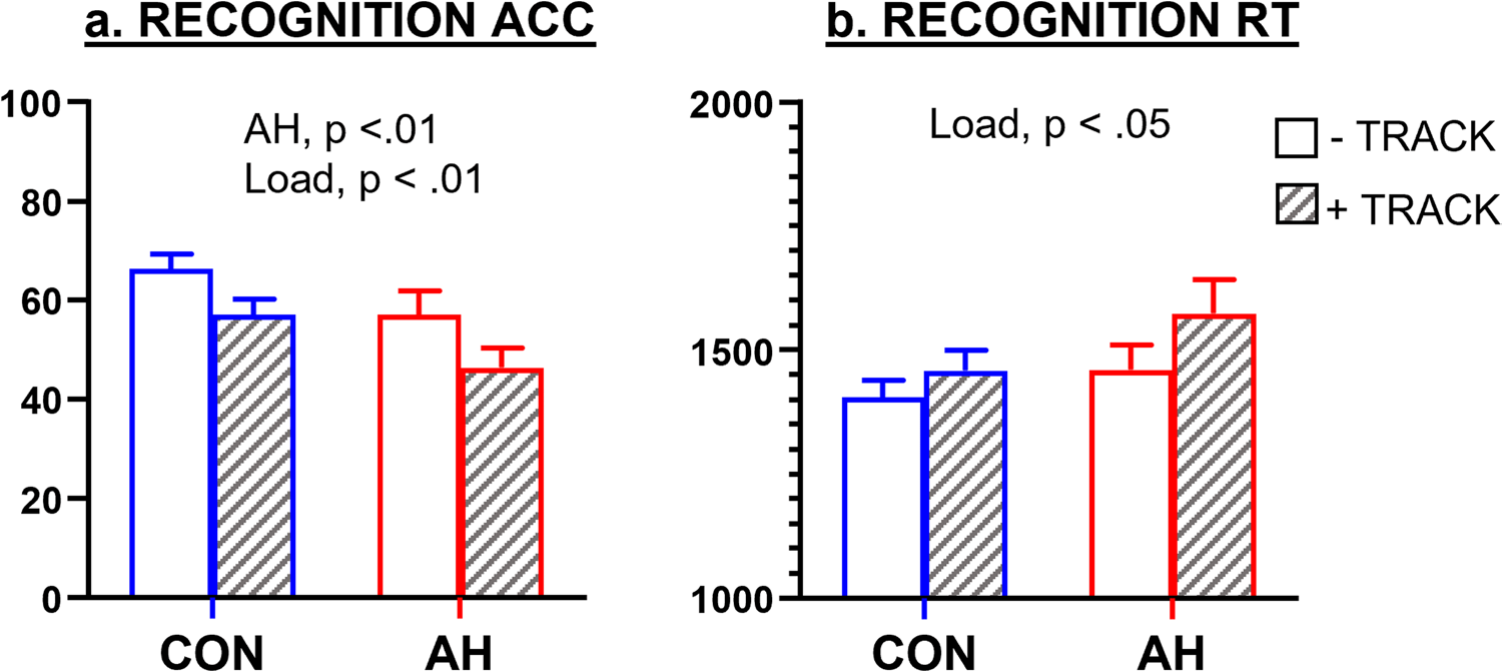
The effects of hangover and load on **a** recognition accuracy (%) and **b** reaction time (msec). Mean (± SEM) number of words correctly recognized during single (- TRACK) and dual-attention (+ TRACK) conditions

Figure 2 displays results for memory recognition. As shown in Fig. 2a, significant main effects of hangover (*F*(1,24) = 12.18, *p* < 0.01, *f* = 0.62) and load (*F*(1,24) = 9.13, *p* < 0.01, *f* = 0.71) were found for delayed recognition accuracy, but there was no hangover × load interaction (*F*(1,24) = 0.036, *p* = 0.85). Further analysis revealed significantly poorer accuracy during AH (62% ± 10.79) than control conditions (67% ± 9.67) for “no” responses (*t*(24) = 3.13, *p* < 0.01, *d* = 0.55, 95% CI [1.87, 9.12]), indicating higher false negative responding. There was no difference in false positives (accuracy for “yes” responses) between AH (72% ± 17.71) and control (77% ± 14.80) (*t*(24) = 1.93, *p* = 0.07, 95% CI [− 0.40, 12.12]) or accuracy for distractor words (*t*(24) = 0.79, *p* = 0.44, 95% CI [− 3.54, 7.94]).

For recognition reaction time (Fig. 2b), there was a significant main effect of Load (*F*(1,24) = 7.92, *p* < 0.05, *f* = 0.57) but not of hangover (*F*(1,24) = 3.71, *p* = 0.07) nor load × hangover interaction (*F*(1,24) = 0.67, *p* = 0.42).

### Perceived performance

**Fig. 3:**
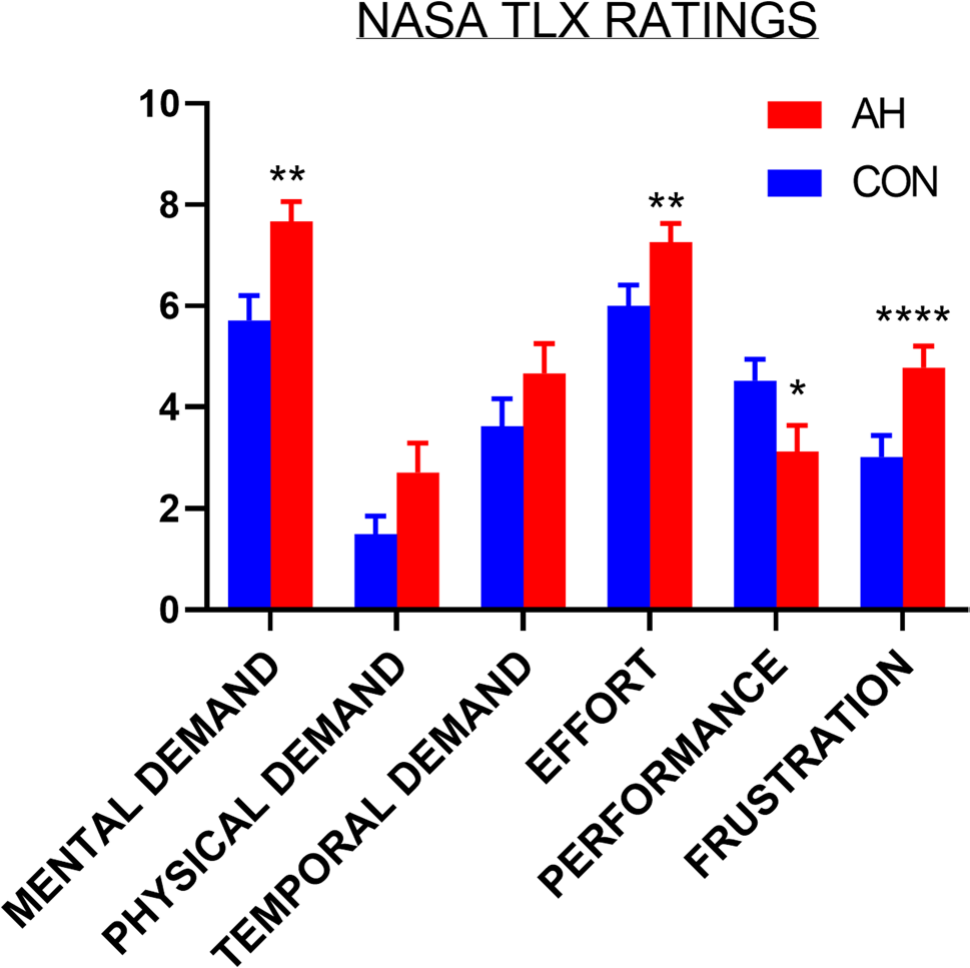
Mean (standard error) perceived levels of workload on the 6 dimensions of the NASA-TLX in AH and control (CON) conditions. *p = .02, **p < .01, ****p < .001

Perceived workload on the NASA-TLX is presented in Fig. 3. Workload ratings on the dual-attention task were significantly higher during AH with large sized effects for mental demand (*t*(24) = 3.55, *p* < 0.01, *d* = 0.85, BCa 95% CI [0.90, 3.13]) and frustration (*t*(24) = 4.29, *p* < 0.001, *d* = 0.76, 95% CI [0.91, 2.61]). Perceived effort was also higher (*t*(24) = 2.96, *p* < 0.01, *d* = 0.66, 95% CI [0.38, 2.15]), and performance was rated as lower during AH (*t*(24) = 2.74, *p* < 0.02, *d* = 0.61, 95% CI [0.35, 2.46]). There was no significant difference in physical (*t*(24) = 2.28, *p* = 0.06, *d* = 0.51, BCa 95% CI [0.30, 2.40]) and temporal demand (*t*(24) = 1.83, *p* = 0.08, *d* = 1.38, 95% CI [− 0.13, 2.21]).

### Correlational analyses

For ease of interpretation and to minimize the risk of type 1 error, only significant variables were considered. AH severity was represented by mAHSS and for task performance, and overall DATT-tracking/encoding scores (i.e., one for words present and one for the interstimulus interval) were calculated. Bivariate correlational analyses revealed an association between AH severity and perceived mental demand (*r* = 0.54, *p* < 0.01), effort (*r* = 0.56, *p* < 0.01), and frustration (*r* = 0.49, *p* < 0.05), but not performance (*p* = 0.21). With the exception of a positive correlation between perceived frustration and the interstimulus interval on the DATT (*r* = 0.47, *p* < 0.05), there was no other evidence that AH severity or perceived workload was associated with DATT performance (*ps* > 0.12) or recognition accuracy (*ps* > 0.06).

## Discussion

The current study explored the effects of AH on attentional resources, memory recognition, and mental effort following a typical night drinking alcohol, compared with a night of no alcohol. As hypothesized, significantly higher tracking costs were found during AH compared with the control condition, indicating potential reduced attentional resources. Decreased recognition accuracy was also found during AH, but the effect was independent of divided attention conditions. Again consistent with our hypotheses, AH resulted in greater effort, frustration, and mental demand as well as poorer perceived performance relating to the DATT. However, limited associations were found between AH severity, task performance, and perceived workload.

No other AH study has compared the effects of AH on divided attention abilities using the dual-attention paradigm. However, increased tracking costs found during AH on the DATT show a similarity with other measures of complex task performance (Yesavage et al. 1994; Zink et al. 2018) including multi-tasking ability (Benson et al. 2020) and driving (Alford et al. 2020a; Verster et al. 2014). The findings are also in contrast from studies measuring visual divided attention (Collins and Chiles 1980; Devenney et al. 2019; Finnigan et al. 2005; Lemon et al. 1993; Roehrs et al. 1991). This indicates that attentional resources may be depleted during an AH, which could impair cognitive processes in situations where attention is divided or there are competing demands.

Unlike previous divided attention research, the current study utilized a paradigm where attention was divided by differing modalities. Increased tracking costs occurred towards the later stages of each word presentation (700–900 ms) and during the interstimulus interval (2200–2400 ms) of a 3000 ms cycle. This is an interesting outcome when compared with other studies using the same paradigm. Scholey et al. (2008) found an increase in tracking costs between 300–1300 ms when words were presented, but no secondary peak. With this in mind, the current findings could be interpreted as AH causing an effect on encoding processes, triggering a subsequent peak in tracking costs due to ongoing memory consolidation requirements. The temporal aspects of these effects need further exploration.

Alternatively, a secondary peak in tracking costs could be related to mental fatigue accrued by high cognitive loads and prolonged attentional demands (Gunn et al. 2018). This was found previously in studies measuring psychomotor speed in relation to sustained attention (Alford et al. 2020b; Howland et al. 2010; Rohsenow et al. 2010). However, as tracking performance alone (i.e., without divided attention conditions) was not recorded, the specific effect of AH on psychomotor performance and how this may have contributed to the peak in tracking costs is unclear from the present results. This should also be considered in future research.

Increased attentional load impaired the speed and accuracy of word recognition. This finding replicated previous studies and is consistent with the concept that concomitant tracking depletes attentional resources (Anderson et al. 2000; Naveh-Benjamin et al. 2005; Scholey et al. 2008). AH also reliably reduced memory recognition accuracy, consistent with previous AH studies (McKinney and Coyle 2004; 2007; Verster et al. 2003). A separate analysis of “yes” and “no” responses revealed differential effects of AH on false negatives, that is responding “no” to a previously encountered word. This finding, coupled with the tracking cost effects, is consistent with the conclusion that AH differentially impairs memory consolidation (Gunn et al. 2018).

Contrary to our hypothesis, there was no interaction between AH and attentional load on memory recognition. This is broadly consistent with our previous findings showing that while AH significantly reduced mood, this effect was not differently affected by engaging in mentally taxing multitasking (Benson et al. 2020). Effect sizes for recognition accuracy were similar for AH and load, though for recognition reaction times, they were greater for load than AH (with AH showing no effect on speed). Overall, recognition scores were low regardless of condition (e.g., only 66% accuracy in no-AH, -tracking condition) compared to other research using the same task (Anderson et al. 2000; Scholey et al. 2008). This may be explained by the cognitive battery presented between phases of the DATT, which was designed to maximize cognitive load and induce mental strain through exposure to multiple stressors simultaneously (Scholey et al. 2014; Wetherell et al. 2012). Therefore, when recognition was assessed following this task, cognitive fatigue induced from this testing may have caused an unintentional effect on memory recognition.

It is worth noting that the lack of interaction between cognitive load and AH could also signify that AH is not modifying attentional resource capacity. Instead, AH may be acting on attentional allocation processes, which consequently interferes with memory recognition. This is largely consistent with previous research that found faster processing of visual information during AH (Stock et al. 2017), as well as an increase in the processing of task-irrelevant information during AH compared with control conditions (Opitz et al. 2020).

The results for perceived workload on the NASA-TLX provided supporting evidence for depleted attentional resources and increased cognitive loads experienced during AH (Gunn et al. 2020). AH significantly increased mental demand, effort, and frustration with medium to large-sized effects similar to previous research (Benson et al. 2020; Verster et al. 2014). Participants also rated their performance as poorer and, despite this awareness, were unable to compensate for impaired functioning. This is different from alcohol intoxication where impairment can occur in the absence of awareness of that impairment (Tiplady et al. 2004).

On the other hand, although AH severity positively correlated with three factors on the NASA-TLX, there was no other evidence of a relationship between AH severity, mental workload, and task performance. Recent research has been mixed regarding the relationship between AH severity and performance, with some studies reporting select associations (Alford et al. 2020a; Ayre et al. 2021) and others not (Gunn et al. 2020, 2021). We previously found correlations between total AH severity and the severity of individual symptoms (e.g., fatigue) with times to complete the trail-making task B (a measure of psychomotor speed and cognitive flexibility). However, no associations were noted for the accuracy of performance (Ayre et al. 2021). These differential effects on cognitive domains may explain some differences observed here.

The findings in this study support growing evidence that AH is associated with reduced attentional resources and poor memory recognition. Impairments found on the DATT in particular could also have real-world implications. For example, the use of in-vehicle verbal navigational systems and hands-free phone devices while driving are common scenarios that engage verbal encoding under already complex divided attention situations. The use of these advanced technologies while driving can also increase the risk of vehicle incidents (Charlton 2009; Cunningham et al. 2017; Lipovac et al. 2017). Considering AH is already associated with poorer driving ability, these findings highlight the even higher risk of driving while experiencing AH (Alford et al. 2020a; Verster et al. 2014).

In terms of study strengths, all participants indicated the presence of AH on the AHSS (see Benson et al. (2020)) and single-item AH scale. The use of a semi-naturalistic research design was also advantageous as the results reflected the effects of “real-life” drinking experiences on cognitive impairment. One limitation of seminaturalistic research is the reliance on self-reported alcohol consumption, which is subject to recall bias and effects eBAC calculations (Devenney et al. 2019; Stephens et al. 2014). Nevertheless, eBAC calculations were reasonably above the threshold for AH and similar to recent experimental studies (Verster et al. 2014, 2020a).

In terms of further limitations, the average standard drinks consumed in this study were lower than comparable seminaturalistic studies in the UK (Alford et al. 2020b). However, this could be due to some participants only consuming a few drinks the previous evening (i.e., range = 3–18.5 drinks) and the higher proportion of females in the study. Alternatively, some cross-cultural research has indicated that alcohol consumed on typical and heavy drinking occasions was comparably lower in Australia, which may explain this discrepancy (Benson et al. 2021).

## Conclusion

The findings in the current study indicated that attentional resources were depleted during AH, which could impair cognitive processing when attention is divided or there are competing demands. AH impaired processes involved with memory encoding while interfering with divided attention performance. AH also reduced memory recognition accuracy but not response speed although this effect was not worse in divided attention conditions suggesting AH resulted in “floor” performance effects. The findings highlight the potential increased risks associated with behaviors such as driving a vehicle and using advanced technology while experiencing AH. Future research should also consider the individual influence of AH on psychomotor performance and immediate memory recognition to discern any differences from dual-attention conditions.
